# Arecoline Promotes Migration of A549 Lung Cancer Cells through Activating the EGFR/Src/FAK Pathway

**DOI:** 10.3390/toxins11040185

**Published:** 2019-03-28

**Authors:** Chih-Hsiang Chang, Mei-Chih Chen, Te-Huan Chiu, Yu-Hsuan Li, Wan-Chen Yu, Wan-Ling Liao, Muhammet Oner, Chang-Tze Ricky Yu, Chun-Chi Wu, Tsung-Ying Yang, Chieh-Lin Jerry Teng, Kun-Yuan Chiu, Kun-Chien Chen, Hsin-Yi Wang, Chia-Herng Yue, Chih-Ho Lai, Jer-Tsong Hsieh, Ho Lin

**Affiliations:** 1 Department of Life Sciences, National Chung Hsing University, Taichung 40227, Taiwan; milkpa@gmail.com (C.-H.C.); atheism1989@gmail.com (T.-H.C.); lihsuancc@gmail.com (Y.-H.L.); sl91123j@yahoo.com.tw (W.-C.Y.); dowdow4820@gmail.com (W.-L.L.); muhammet.oner053@gmail.com (M.O.); 2 Medical Center for Exosomes and Mitochondria Related Diseases, China Medical University Hospital, Taichung 40447, Taiwan; midyjack@gmail.com; 3 Department of Nursing, Asia University, Taichung 41345, Taiwan; 4 Department of Applied Chemistry, National Chi Nan University, Nantou 54561, Taiwan; ctyu@ncnu.edu.tw; 5 Institute of Medicine, Chung Shan Medical University, Taichung 40201, Taiwan; daniel@csmu.edu.tw; 6 Division of Chest Medicine, Taichung Veterans General Hospital, Taichung 40705, Taiwan; jonyin@gmail.com (T.-Y.Y.); ckjohn@mail.vghtc.gov.tw (K.-C.C.); 7 Division of Hematology/Medical Oncology, Taichung Veterans General Hospital, Taichung 40705, Taiwan; drteng@vghtc.gov.tw; 8 Division of Urology, Taichung Veterans General Hospital, Taichung 40705, Taiwan; chiu37782002@yahoo.com; 9 Department of Nuclear Medicine, Taichung Veterans General Hospital, Taichung 40705, Taiwan; hywang@vghtc.gov.tw; 10 Department of Surgery, Tung’s Taichung Metro Harbor Hospital, Taichung 435, Taiwan; ericchyue@gmail.com; 11 Department of Microbiology and Immunology, Chang Gung Medical University, Taoyuan 33302, Taiwan; chlai@mail.cgu.edu.tw; 12 Department of Urology, University of Texas Southwestern Medical Center, TX 75390, USA; JT.Hsieh@UTSouthwestern.edu; 13 Rong Hsing Research Center for Translational Medicine, National Chung Hsing University, Taichung 40227, Taiwan; 14 Ph.D. Program in Translational Medicine and Rong Hsing Research Center for Translational Medicine, National Chung Hsing University, Taichung 40227, Taiwan

**Keywords:** Arecoline, lung cancer cells, mAchR3, EGFR, SRC, FAK

## Abstract

Arecoline is the primary alkaloid in betel nuts, which are known as a risk factor for oral submucosal fibrosis and oral cancer. Lung cancer is a severe type of carcinoma with high cell motility that is difficult to treat. However, the detailed mechanisms of the correlation between Arecoline and lung cancer are not fully understood. Here, we investigated the effect of Arecoline on migration in lung cancer cell lines and its potential mechanism through the muscarinic acetylcholine receptor 3 (mAChR3)-triggered EGFR/Src/FAK pathway. Our results indicate that different concentrations of Arecoline treatment (10 µM, 20 µM, and 40 µM) significantly increased the cell migration ability in A549 and CL1-0 cells and promoted the formation of the filamentous actin (F-actin) cytoskeleton, which is a crucial element for cell migration. However, migration of H460, CL1-5, and H520 cell lines, which have a higher migration ability, was not affected by Arecoline treatment. The EGFR/c-Src/Fak pathway, which is responsible for cell migration, was activated by Arecoline treatment, and a decreased expression level of E-cadherin, which is an epithelial marker, was observed in Arecoline-treated cell lines. Blockade of the EGFR/c-Src/Fak pathway with the inhibitors of EGFR (Gefitinib) or c-Src (Dasatinib) significantly prevented Arecoline-promoted migration in A549 cells. Gefitinib or Dasatinib treatment significantly disrupted the Arecoline-induced localization of phospho-Y576-Fak during focal adhesion in A549 cells. Interestingly, Arecoline-promoted migration in A549 cells was blocked by a specific mAChR3 inhibitor (4-DAMP) or a neutralizing antibody of matrix metalloproteinase (MMP7 or Matrilysin). Taken together, our findings suggest that mAChR3 might play an essential role in Arecoline-promoted EGFR/c-Src/Fak activation and migration in an A549 lung cancer cell line.

## 1. Introduction

More than a million people in Taiwan consume betel nuts, and betel nut chewing is known as a risk factor for oral cancer and oral submucosal fibrosis, as described by the International Agency for Research on Cancer (IACR) [1,2,3]. Feeding Swiss mice and C17 mice a betel nut extraction induces gastrointestinal tumors in 58% and 17%, respectively. Studies have shown that betel nut extraction with DMSO, given for 21 weeks, increased tumor formation in the oral mucosa and caused leukoplakia in hamsters [4,5]. A tumorigenicity study in Swiss mice showed that betel nuts and betel quid induced lung cancer formation (47% and 26%, respectively) [6]. This data suggests that the habit of chewing betel nuts not only increases the risk of head and neck cancer, but also elevates the systemic risk of carcinogenesis. Arecoline is a primary alkaline in betel nut extract, and is also detected in the saliva of people who chew betel nuts, at a concentration between 5.66 and 97.39 μg/mL (approximately 0.036 mM to 0.63 mM) [5,7], and about 7 ng/mL (approximately 0.044 μM) in peripheral blood plasma [8]. Cytological studies indicate that a higher Arecoline level (more than 0.4 mM) can cause cytotoxicity in keratinocytes, and DNA damage when used long-term [9].

Arecoline is an acetylcholine agonist that acts on the neuronal nicotinic acetylcholine receptor (nAChR) or the muscarinic acetylcholine receptor (mAChR), which are G protein-coupled receptors in the cell membrane. It has been considered that Arecoline, as an acetylcholine agonist, might be useful in neurodegenerative diseases, such as Alzheimer’s disease [10]. In the previous study, betel quid extract was found to promote cell migration in an esophageal squamous carcinoma cell line CE81T/VGH, and purified Arecoline treatment also had a similar effect [11], while matrix metalloproteinase (MMP) expression, specifically MMP-1 and MMP-8, increased with Arecoline treatment. Increased cell motility was inhibited by MMP-1 [12] or MMP-8 [11] neutralizing antibody, suggesting that activation of MMPs may be involved in Arecoline-stimulated migration. Studies on human colon cancer cells have shown that after ligand binding to muscarinic acetylcholine receptor 3 (mAChR3; M3R), MMP7 was activated, with subsequent release of the EGFR ligand [13,14]. mAchR3-transactivated EGFR downstream signaling pathways, including Erk1/2 [14], PI3K/Akt [13,15] and FAK/c-Src [16,17], are involved in cell survival and migration. The activation of mAChR3 is known as a multiplying factor in non-small cell lung cancer. In a previous clinical study, 85 patients, from 148 studied patients (57.4%), had high expression of mAChR3 in paraffin-embedded non-small cell lung cancer (NSCLC) tissue samples, which correlated well with tumor metastasis and poor survival [18,19]. Down-regulation of mAChR3, by siRNA, inhibited the migration and invasion ability of the lung cancer cell lines A549 and L78 [18]. Activation of mAChR3 in A549 cells also induced epithelial–mesenchymal transition (EMT) marker expression [19]. These studies showed that over-expression or activation of mAChR3 promotes the progression of lung cancer.

According to previous studies, acetylcholine promoted lung cancer cell proliferation and migration through the mAChR3-activated EGFR/PI3K/Akt pathway accompanied by partial MMP activation [13,14,15]. Arecoline, an agonist of acetylcholine, also activated MMPs and was involved in esophageal carcinoma cell migration [11,12]. Thus, we suggest that Arecoline-promoted lung cancer cell migration is mediated by MMP activation; however, the detailed mechanism has not been determined. We hypothesize that administration of Arecoline contributes to lung cancer cell migration through the EGFR/c-Src/FAK pathway via mAChR3 transactivation. Cell motility was measured by the cell migration assay, which is a standard in vitro technique for detecting cell migration, and the activation of EGFR, c-Src and FAK were analyzed. We further used inhibitors or antagonists of mAChR3, EGFR, c-Src and neutralizing antibodies of MMP7 to verify the pathway. In this study, we suggest that Arecoline consumption and activation of mAChR3 is positively correlated with lung cancer cell migration, and interrupting this potential pathway may lead to decreased lung cancer cell migration.

## 2. Results

### 2.1. Arecoline Promotes Migration in the A549 Lung Cancer Cell Line

**Figure 1:**
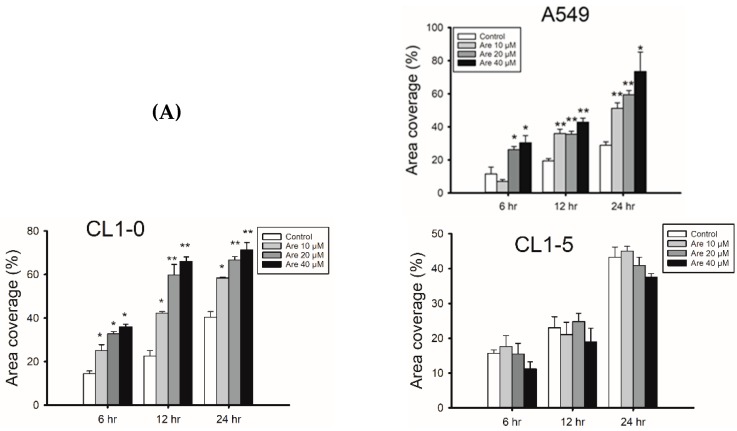

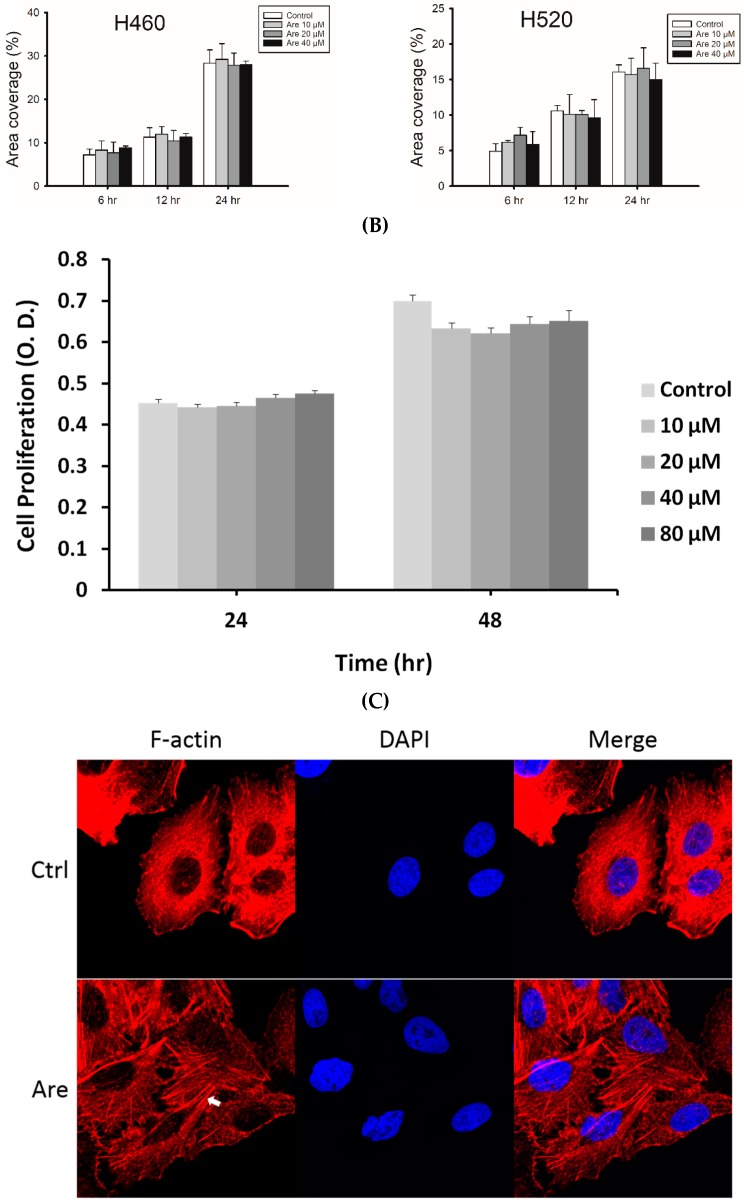
Arecoline promotes A549 and CL1-0 lung cancer cell migration. (**A**) Cell motility was measured by the cell migration assay. Lung cancer cells were seeded in an ibidi Culture-Insert with a cell density of 5 × 10^4^ cells/well. Cell images were taken at indicated time points after treatment with a variable concentration of Arecoline. The results show that Arecoline promoted lung cancer cell migration in A549 and CL1-0 cells, but not in H520, H460, and CL1-5 cells. (*: *p*< 0.05; **: *p* < 0.01, compared with control groups). (**B**) The MTT assay was performed to detect cell viability. Different concentrations of Arecoline were administrated to A549 cells, and cell proliferation was measured at 24 and 48 h. (**C**) A549 cells treated with 40 μM Arecoline for 24 h and then fixed for immunostaining. F-actin was stained with phalloidin and DAPI for nuclear staining.

In this study, we suggest that Arecoline activates the EGFR and c-Src/FAK signaling cascade to stimulate cell migration through regulation of mAChR3. The activation of the signal cascade was analyzed by immunoblotting of active EGFR, c-Src and FAK, and the distributions of activated proteins were observed by fluorescent confocal microscopy. We also used inhibitors of EGFR, c-Src and FAK to confirm the pathway. In the first step, different concentrations of Arecoline (Are) were administrated to several lung cancer cell lines and cell motility was measured. The covered area was measured and quantified at 0, 6, 12 and 24 h after Arecoline treatment. The results indicate that administration of Arecoline stimulated A549 and CL1-0 cell migration in a dose-dependent manner, but not in H520, H460 and CL1-5 cells (Figure 1A). After 24 h of Arecoline treatment, the coverage of A549 and CL1-0 cells significantly increased to around 50% with 10 μM, 60% with 20 μM and 70% with 40 μM Arecoline compared with the control groups. However, the coverage of H520, H460, and CL1-5 cells did not change compared with control groups in all treatments. The MTT assay showed that there was no increase in cell proliferation in Arecoline-treated A549 cells (Figure 1B). The viability results of CL1-0, CL1-5, H520 and H460 after Arecoline treatment are also provided in Figure S1. This result suggests that Arecoline did not affect cell proliferation, but it did cause an increased coverage of A549 cells. The immunofluorescent study showed that stress fiber formation increased in Arecoline-treated A549 cells (Figure 1C). This means that Arecoline stimulates a morphological change and leads to the migration of lung cancer cells. Regarding the response of non-cancerous cells, MRC-5, no apparent differences in viability and migration were observed after Arecoline treatment (Figure S2), which indicates that the action of Arecoline is specific to cancer cells.

### 2.2. Arecoline Treatment Activates the EGFR/c-Src/FAK Signaling Pathway in the A549 Cell Line

**Figure 2:**
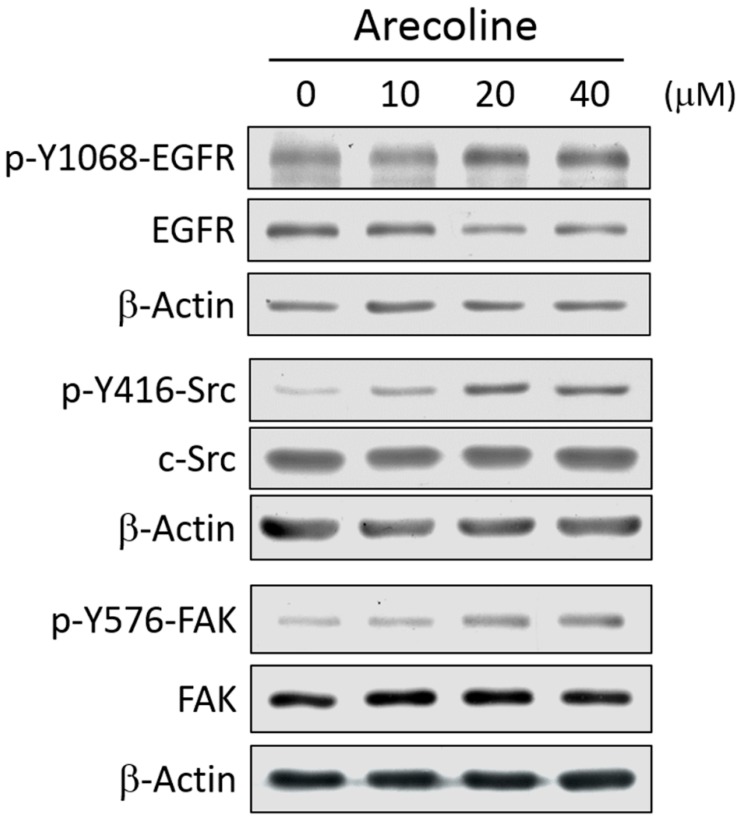
Arecoline activates EGFR/c-Src/FAK in a dose-dependent manner. After treatment with 10, 20 or 40 μM Arecoline for 24 h, A549 cells were collected, and proteins were analyzed by immunoblotting. The expression and phosphorylation of EGFR (pY1068-EGFR), c-Src (pY416-Src) and FAK (pY397-FAK) were measured. Actin served as an internal control.

After Arecoline treatment at different concentrations, A549 cells were collected, and the total proteins were extracted and separated by SDS-PAGE and the activation of EGFR, c-Src, and FAK was measured by immunoblotting. Their phosphorylation antibodies determined the activation of EGFR, c-Src, and FAK at pY1068, pY416 and pY576, respectively (Figure 2). The result showed that the phosphorylation levels of pY1068-EGFR, pY416-c-Src and pY576-FAK increased in a dose-dependent manner with Arecoline treatment. The highest activation levels of EGFR, c-Src and FAK were observed with 40 μM Arecoline treatment for 24 h. The effects of Arecoline on Src and FAK in CL1-0, H520 and H460 are also provided in Figure S3.

### 2.3. Arecoline Stimulates Epithelial–Mesenchymal Transition (EMT) Markers in the A549 Cell Line

**Figure 3:**
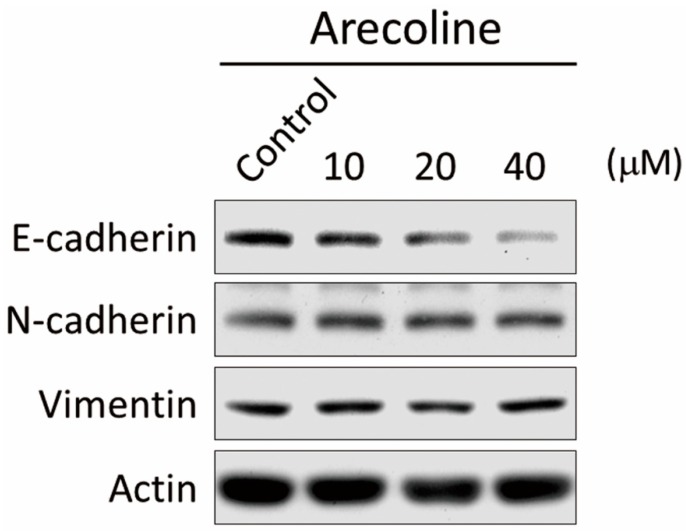
Arecoline administration induced epidermal–mesenchymal transition (EMT) marker expression in A549 cells. A549 cells were treated with 10, 20 or 40 μM Arecoline for 24 h and the protein expression of E-cadherin, N-cadherin, and vimentin was analyzed.

A549 cells were treated with different concentrations of Arecoline for 24 h and the expression of E-cadherin, N-cadherin, and vimentin were analyzed. The results indicated that the expression of E-cadherin decreased in Arecoline-treated A549 cells in a dose-dependent manner, while N-cadherin and vimentin were not affected (Figure 3).

### 2.4. The Inhibition of EGFR or c-Src Activation Reversed Arecoline-Stimulated Migration in the A549 Cell Line

**Figure 4:**
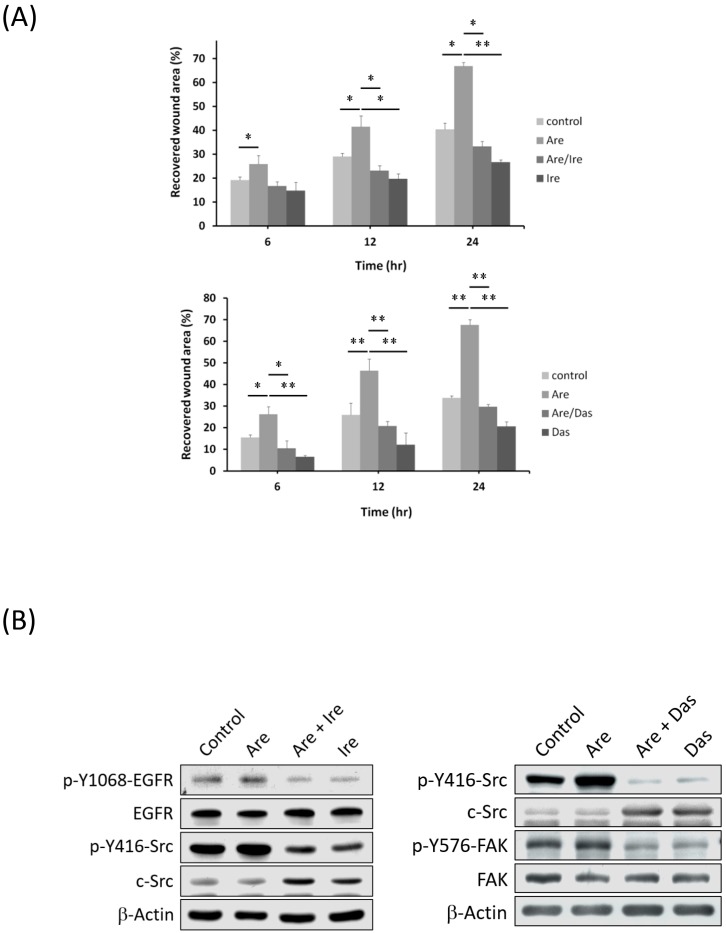

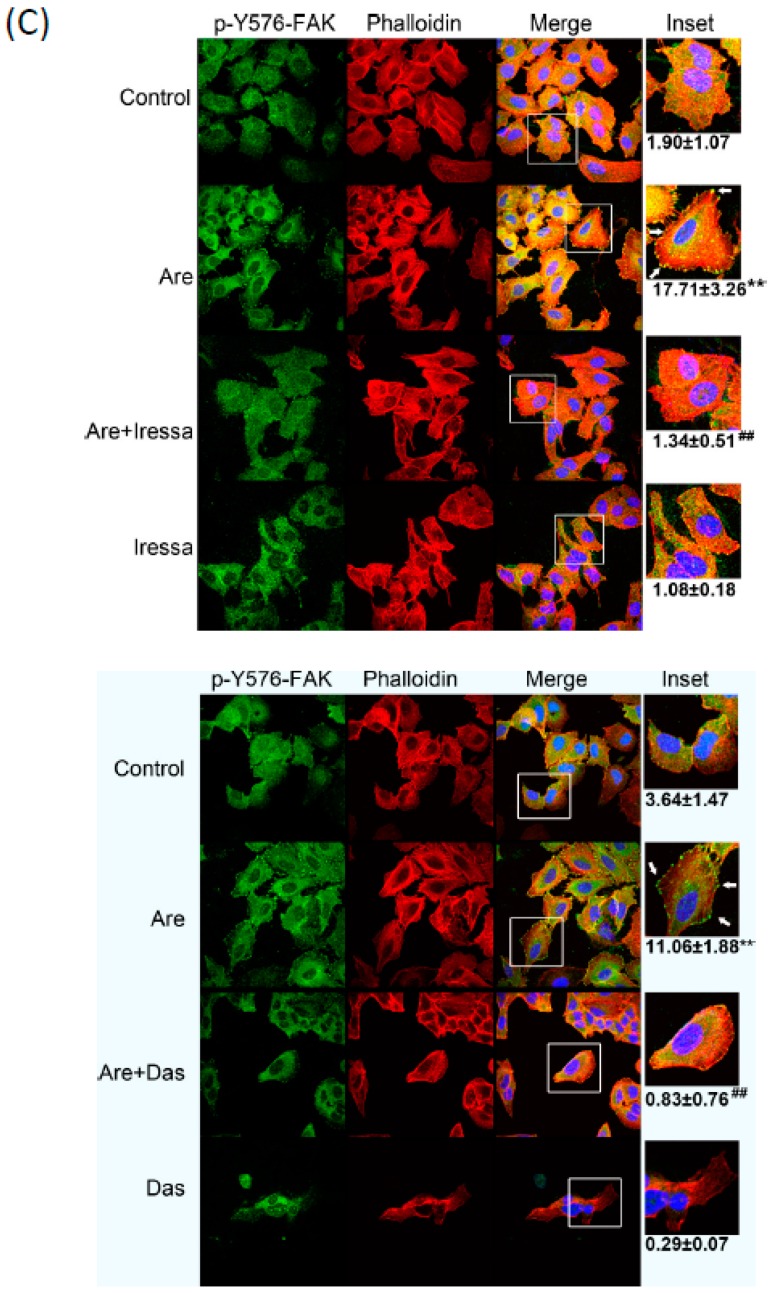
Gefitinib and Dasatinib reversed Arecoline-induced A549 cell migration and signaling activation. A549 cells were co-treated with 40 μM Arecoline (Are) and 50 μM Gefitinib (Gef) for 24 h. (**A**) Cell motility was recorded at indicated time points, and the result showed that Arecoline-stimulated cell migration was reversed by co-administration of Gefitinib or Dasatinib. (**B**) Protein expression and phosphorylation were measured by immunoblotting. The results showed that Arecoline-stimulated EGFR and c-Src activation reduced after Gefitinib or Dasatinib treatments. (*: *p* < 0.05; **: *p* < 0.01, compared with control groups). (**C**) Phosphorylated pY576-FAK (green), F-actin (red) and cell nucleus (blue) were detected by immunofluorescence. Activated FAK was observed in focal adhesion after Arecoline treatment, and counted. Numbers show an average activated FAK per cell (**: *p* < 0.01, compared with control groups; ^##^: *p* < 0.01, compared with Arecoline groups); however, the accumulation of activated FAK was reduced by Gef or Das treatments.

Immunoblotting and the cell migration assay showed that Arecoline stimulates A549 lung cancer cell migration. The activation of the EGFR/c-Src/FAK signaling pathway was investigated. Arecoline-stimulated migration reversed through inhibition of EGFR or c-Src activity by co-administrating 50 μM Gefitinib (Gef) or 50 nM Dasatinib (Das). The cell migration assay showed that co-administration of Gefitinib (Gef) or Dasatinib reversed A549 cell migration (Figure 4A). Furthermore, the subsequent decrease in c-Src and FAK by Gef or Das was detected by immunoblotting. After co-treatment with Arecoline and Gef or Das for 24 h, proteins were collected and analyzed by immunoblotting. The expression levels of EGFR, c-Src or FAK reduced in the Gef or Das co-treated groups (Figure 4B). Arecoline-stimulated activation of EGFR, c-Src and FAK was reversed by Gef or Das. Furthermore, the distribution of phosphorylated FAK (pY576-FAK) was also investigated by immunofluorescent staining. Accumulation of pY576-FAK showed that focal adhesion formation, as part of cell migration, was induced by Arecoline. The results indicated that pY576-FAK accumulated after Arecoline treatment for 24 h (Figure 4C). Accumulated pY576-FAK signals in Arecoline-treated cells were counted and increased 3 to 8 fold (11.06/3.64 to 17.71/1.90). Accumulation of pY576-FAK was induced by Gef and Das treatments (Figure 4C).

### 2.5. Arecoline Stimulates Lung Cancer Cell Migration through the Muscarinic Acetylcholine Receptor 3 (mAChR3) Transactivating EGFR Pathway in the A549 Cell line

**Figure 5:**
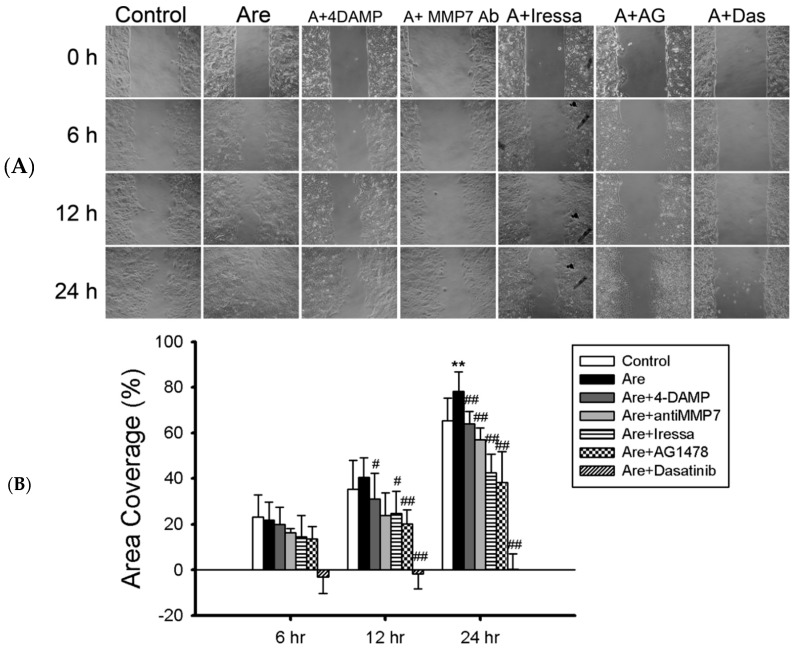
Muscarinic acetylcholine receptor 3-dependent A549 cell migration by Arecoline stimulation. (**A**) Cell migration assays were performed and A549 cells treated with Arecoline 40 μM (Are) were co-administrated with one of the following reagents: mAChR3 inhibitor (4-DAMP) 10 μM (Are+4-DAMP); MMP7 neutralizing antibody (MMP7 Ab) 2 μg/mL (Are+MMP7 Ab); EGFR inhibitor: Gefitinib 50 μM (Are+Gefitinib)/ AG1478 (AG) 50 μM (Are+AG); c-Src inhibitor Dasatinib (Das) 50 nM (Are+Das). (**B**) The results of cell motilities were quantified and compared with control or Arecoline treatments. (**: compared with control group, *p* < 0.01; ^#^: compared with Arecoline-treated (Are) group, *p* < 0.05; ^##^: compared with Arecoline-treated (Are) group, *p* < 0.01).

The hypothesis is that Arecoline stimulates A549 lung cancer cell migration by mAChR3 transactivating EGFR and the following c-Src/FAK signaling pathway. Increased coverage area, stimulated by Arecoline administration, was observed, and then inhibitors that block the hypothesized pathway were further applied. After co-administration of 10 μM 4-DAMP (mAChR3 antagonist), 50 μM Gefitinib, 50 μM AG-1478 (EGFR inhibitors), 50 nM Dasatinib (c-Src inhibitor) or 2 μg/mL anti-MMP7 neutralizing antibody, Arecoline-stimulated migration recovered significantly in all treatment groups (Figure 5).

## 3. Discussion

In Taiwan, more than a million people chew betel nuts. Betel chewing has been considered a risk factor for oral cancer and oral submucosal fibrosis; furthermore, animal studies have also shown that betel nuts can induce gastrointestinal tumors or lung cancer in Swiss mice [4,6]. These data suggest that betel-chewing habits increase not only carcinogenic risk in the oral cavity but also in the digestive tract and lungs. Furthermore, previous studies have shown that activation of muscarinic acetylcholine receptor 3 (mAChR3) promoted non-small cell lung cancer metastasis and invasion [15,18]. In this study, lung cancer cells were used, and Arecoline stimulated lung cancer cell migration. Arecoline stimulated cell migration in A549 and CL1-0 cells, but not in H460, H520, and CL1-5 cells. H520 and H460 cell lines have lower EGFR expression compared with other lung cancer cell lines, such as A549 or CL1-0 [20,21], which may be the reason why Arecoline administration did not stimulate their migration. Corresponding to the migration results, Arecoline induced phosphorylation of Src and FAK in CL1-0 cells but not in H520 and H460 cells (Supplementary Figure S3). On the other hand, CL1-5 derived from CL1-0 with higher EGFR expression and invasive ability [22]; meanwhile, CL1-0 and CL1-5 cells showed different downstream responses after EGF-stimulation [23]. The protein levels of phospho-EGFR/EGFR in CL1-5 were relatively high compared to those in CL1-0. Therefore, Arecoline might not further stimulate EGFR activation in CL1-5 when it is already at a high level.

**Figure 6:**
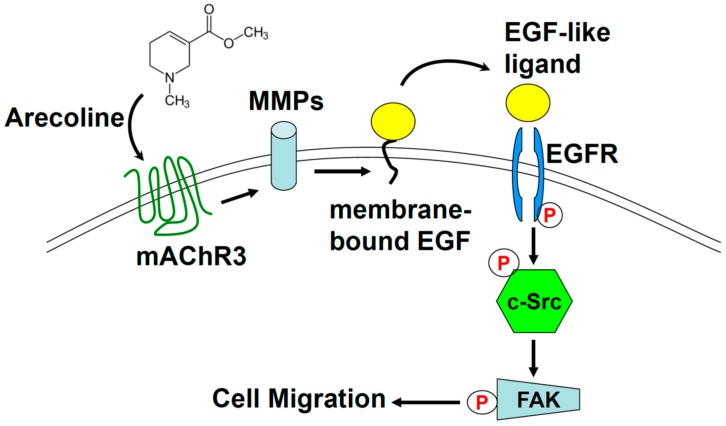
The scheme of the Arecoline-transactivated EGFR/c-Src/FAK pathway through mAChR3 for stimulating lung cancer cell migration. Arecoline stimulates MMP activity via mAChR3 to cleave EGF-like ligand and then subsequently trigger the EGFR/c-Src/FAK signaling cascade that stimulates lung cancer cell migration.

According to the wound healing results, we found that Arecoline-stimulated cell motility can be reversed by co-administrating a mAChR3, EGFR, c-Src inhibitor or MMP-7 neutralizing antibody (Figure 5). Investigation of signaling cascade activation also presented similar results; the introduction of Arecoline elevated the phosphorylation level of EGFR, c-Src and FAK (Figure 2). The formation of focal adhesion and activated FAK accumulation was also observed (Figure 4C). The activated signaling cascade decreased by co-administration of EGFR or a c-Src inhibitor. Based on these data, Arecoline-stimulated A549 lung cancer cell migration through mAChR3 transactivating the EGFR/c-Src/FAK pathway was confirmed. Previous studies showed that mAChR3 activation stimulated cell proliferation, migration, and invasion in colon cancer and small cell lung cancer [13,14,15,24], and also other studies showed that the overexpression of mAChR3 in non-small cell lung cancer cells promoted NSCLC progress and poor prognosis [18,25]. Muscarinic acetylcholine receptor 3 is a member of the G-protein coupled receptor (GPCR) family; the activation of mAChR3 can transactivate EGFR by the MMP-cleaved EGF-like ligand [13,14,15,26,27,28]. Several studies showed that post-EGFR pathways are involved in promoting cell migration, including the MEK/ERK1/2 pathway in colon cancer cells [13,14], or the PI3K/AKT signaling pathway in NSCLC [15]. Receptor tyrosine kinase- (RTK), integrin- or GPCR-activated c-Src contributes to many cellular functions, including cell migration [16,29,30]. In this study, we showed that Arecoline promoted migration through mAChR3 to transactivate the EGFR/c-Src/FAK signaling pathway (Figure 6).

Furthermore, we showed that cell motility increased in Arecoline-treated lung cancer cells in a dose-dependent manner (Figure 1A). This suggests that Arecoline provides a driving force in lung cancer cell migration. Administration of 4-DAMP or anti-MMP7 neutralizing antibody reversed cell motility. This confirms that the transactivating effects were reversed in A549 lung cancer cells by a mAChR3 inhibitor and MMP7 neutralizing antibody [13,14,28,31]. Although Puthenedam’s report showed that MMP7-cleaved galectin-3 inhibited the motility of intestinal epithelial cells [32], there was no further evidence indicating the phenomena could be applied to lung cancer cells. Besides MMP7, activated muscarinic acetylcholine receptors induced other MMPs, such as MMP1 [14,33], MMP3 [26], MMP9 [34] and MMP10 [14] expression, which are involved in cell invasion and migration. Several studies showed that MMP1 [12] and MMP8 [11] expressions were also elevated in esophageal carcinoma cells; however, these studies did not mention the effect of Arecoline on the muscarinic acetylcholine receptor. In this study, we elucidate that Arecoline stimulated cell migration by transactivating EGFR through the mAChR3/MMP7-cleaved EGF-like ligand and then activating the c-Src/FAK signaling pathway (Figure 6). Together, our results establish that Arecoline might be considered as a novel risk factor in NSCLC metastases.

## 4. Materials and Methods

### 4.1. Cell Lines and Cell Culture

Non-small lung cancer cell lines A549 (ATCC number: CCL-185; BCRC number: 60074), and H520 (ATCC number: HTB-182; BCRC number: 60124) were purchased from Food Industry Research and Development Institute, Hsinchu, Taiwan. CL1-0 and CL1-5 cell lines were kindly provided by Professor Jeremy J. W. Chen, Institute of Molecular Biology, National Chung Hsing University. A549 cells were cultured in F-12K medium containing 10% FBS, 1.5 g/L NaHCO_3_, 20 mM L-glutamine and 1% penicillin-streptomycin (P/S); H520, CL1-0, and CL1-5 cells were cultured in RPMI 1640 culture medium supplemented with 1.5 g/liter NaHCO_3_, 10% FBS, 10 mM HEPES, 1 mM sodium pyruvate, and P/S. All cell lines were cultured at 37 °C in a humidified atmosphere with 5% CO_2_.

### 4.2. Cell Viability Assay

Cells were seeded in the 96-well plate and incubated for 24 h after attachment. Cells were treated with different concentrations of Arecoline (Sigma-Aldrich, St. Louis, MO, USA) for the indicated number of days, and then the 3-(4,5-dimethylthiazol-2-yl)-2, 5-diphenyl tetrazolium bromide (MTT, Sigma-Aldrich, St. Louis, MO, USA) assay was used to quantify cell proliferation. The MTT stock solution (5 mg/mL) was diluted to 0.5 mg/mL with complete culture medium, and 0.1 mL MTT working solution was added to each well. Yellow MTT was converted to blue formazan by living cells, a reaction that is dependent on mitochondrial enzyme activity. After using DMSO (J. T. Baker, Center Valley, PA, USA) to dissolve the blue formazan, the absorbance of converted MTT could be measured at 570 nm.

### 4.3. Cell Migration Assay

The day before treatment, 5 × 10^4^ cells/well were seeded into the culture insert (ibidi GmbH, Planegg, Germany) for attachment. After starvation with serum-free medium for 24 h, inserts were removed, and cells were treated with Arecoline and Arecoline co-treated with 10 μM 4-DAMP, 2 μg/mL MMP7 neutralizing antibody, 50 μM Gefitinib, 50 μM AG1478 or 50 nM Dasatinib. Pictures were obtained at the indicated time by optical microscopy (Olympus, CKX41, Tokyo, Japan) and analyzed by TScratch software (1.0, Computational Science & Engineering Laboratory, Swiss Federal Institute of Technology, Zurich, Switzerland, 2010) [35].

### 4.4. Immunoblotting

Treated cells were collected and washed with PBS then the cells were lysed by RIPA buffer (50 mM Tris, 150 mM NaCl, 2mM EDTA, 50 mM NaF, 0.1% SDS, 0.5% sodium deoxycholate and 1% NP-40). Obtained cell lysates were quantified by Bradford reagent (Sigma-Aldrich, St. Louis, MO, USA) and separated by SDS-PAGE (25 μg/lane). After being transferred, PVDF membranes (PerkinElmer Life Sciences, Shelton, CT, USA) were blocked with 5% skim milk and then incubated with primary antibodies overnight at 4 °C. After washing with PBST, horseradish peroxidase (HRP)-conjugated secondary antibodies (Jackson Immuno Research Laboratory, West Grove, PA, USA) were incubated at room temperature. The Enhanced Chemiluminescence (PerkinElmer Life Sciences, Shelton, CT, USA) reaction was performed, and the membranes were exposed to X-ray films (Fujifilm, Tokyo, Japan). Antibodies directed against the following proteins were used in this study: anti-Actin (Millipore, MAB1501, Temecula, CA, USA), anti-Tubulin (Millipore, 05829), anti-GADPH (GeneTex, GTX100118, Irvine, CA, USA), anti-cSrc (Santa Cruz, sc-8056, Dallas, TX, USA), anti-pY416-Src (Cell signaling, 2101), anti-EGFR (Santa Cruz, sc-03), anti-pY1068-EGFR (Cell signaling, 2036, Danvers, MA, USA), anti-E-cadherin (#610181, BD, Franklin Lakes, NJ, USA), anti-Vimentin (Santa Cruz, sc-32322), anti-pY397-FAK (BD, 611723), anti-pY576-FAK (Santa Cruz, sc-16563-R).

### 4.5. Immunostaining

Cells were seeded on a cover-slip the day before treatment; treated cells were washed with PBS and fixed in 3.7% paraformaldehyde (Sigma-Aldrich, St. Louis, MO, USA) for 30 min at room temperature. Followed by washing three times with PBS and blocking with 3% BSA in PBS, cells were incubated with anti-pY576-FAK for 1 h. After washing with PBST, cells were incubated with FITC-conjugated anti-Rabbit secondary antibody for 1 h. For F-actin and nuclear staining, 5 μg/mL phalloidin-TRITC (Sigma-Aldrich, St. Louis, MO, USA) and 1 μg/mL DAPI (Sigma-Aldrich, St. Louis, MO, USA) were applied. After further washing with PBS, slides were mounted for observation by fluorescence microscopy (Olympus, BX51, Japan) and laser confocal microscopy (Olympus, FV1000, Japan).

### 4.6. Statistical Analysis

The data were presented as means ± standard error of the mean (SEM). Student’s *t*-test was used in the cell viability assay, cell migration assay, and immunoblotting. A significant difference between treatments was considered when *p* < 0.05.

## Supplementary Materials

The following are available online at https://www.mdpi.com/2072-6651/11/4/185/s1, Figure S1: The effects of Arecoline on the viability of different lung cancer cell lines. Figure S2: Arecoline does not affect viability and migration of MRC-5 cells. Figure S3: The effects of Arecoline on Src and FAK in CL1-0, H520 (A), and H460 cells (B).

## Abbreviations

EGF	Epithelial growth factor
EGFR	Epithelial growth factor receptor
EMT	Epithelial-mesenchymal transition
GPCR	G-protein coupled receptor
mAChR3	Muscarinic acetylcholine receptor 3
MMPs	Matrix metalloproteinases
RTK	Receptor tyrosine kinase
