# Supplementary material for: Arecoline Promotes Migration of A549 Lung Cancer Cells through Activating the EGFR/Src/FAK Pathway
Source: Toxins (Basel). 2019 Mar 28;11(4):185. doi: 10.3390/toxins11040185 (PMC6521018; doi:10.3390/toxins11040185)
Supplement: Supplementary file 1 [file toxins-11-00185-s001.pdf]

Supplementary Materials: Arecoline promotes migration of A549 lung cancer cells through activating EGFR/Src/FAK pathway

Chih-Hsiang Chang, Mei-Chih Chen, Te-Huan Chiu, Yu-Hsuan Li, Wan-Chen Yu, Wan-Ling Liao, Muhammet Oner, Chang-Tze Ricky Yu, Chun-Chi Wu, Tsung-Ying Yang, Chieh-Lin Jerry Teng, Kun-Yuan Chiu, Kun-Chien Chen, Hsin-Yi Wang, Chia-Herng Yue, Chih-Ho Lai, Jer-Tsong Hsieh and Ho Lin

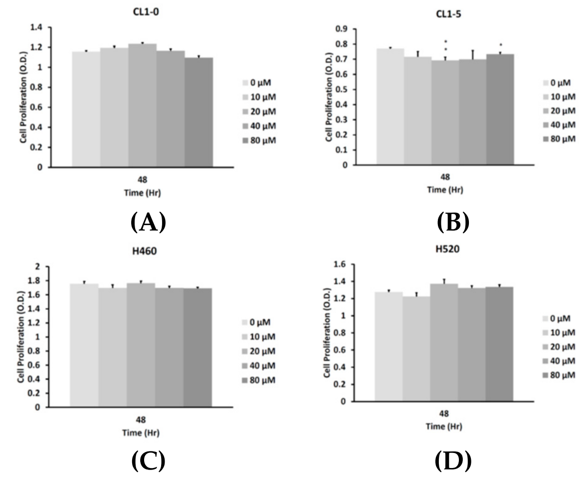

Figure S1. The effects of Arecoline on viability of different lung cancer cell lines. MTT assay was performed to detect cell viability. Different concentrations of Arecoline were administrated to CL1-0 (**A**), CL1-5 (**B**), H460 (**C**), and H520 (**D**) cells and the cell proliferation was measured at 48 hours. (*: $p < 0.05$; **: $p < 0.01$, compared with control groups).

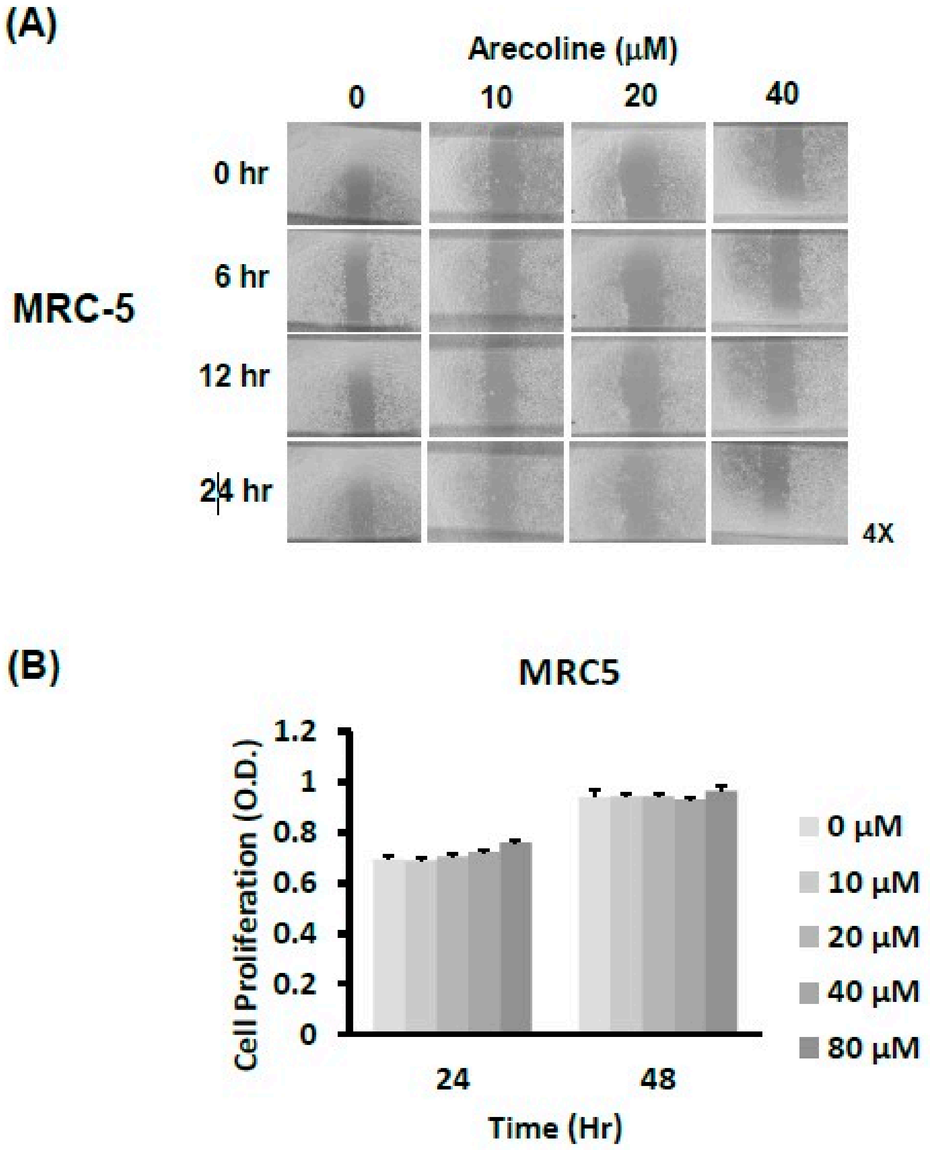

Figure S2. Arecoline does not affect viability and migration MRC-5 cells. (**A**) Cell motility was measured by cell migration assay. MRC-5 normal lung cells were seeded in ibidi Culture- Insert with cell density: 5×104 cells/well. Cell images were taken at indicated time points after treated with a variant concentration of Arecoline. The result shows no apparent response of Arecoline to MRC-5 migration. (**B**) MTT assay was performed to detect cell viability. Different concentrations of Arecoline were administrated to MRC-5 cells and the cell proliferation was measured at 24 and 48 hours. The result shows no apparent response of Arecoline to MRC-5 viability.

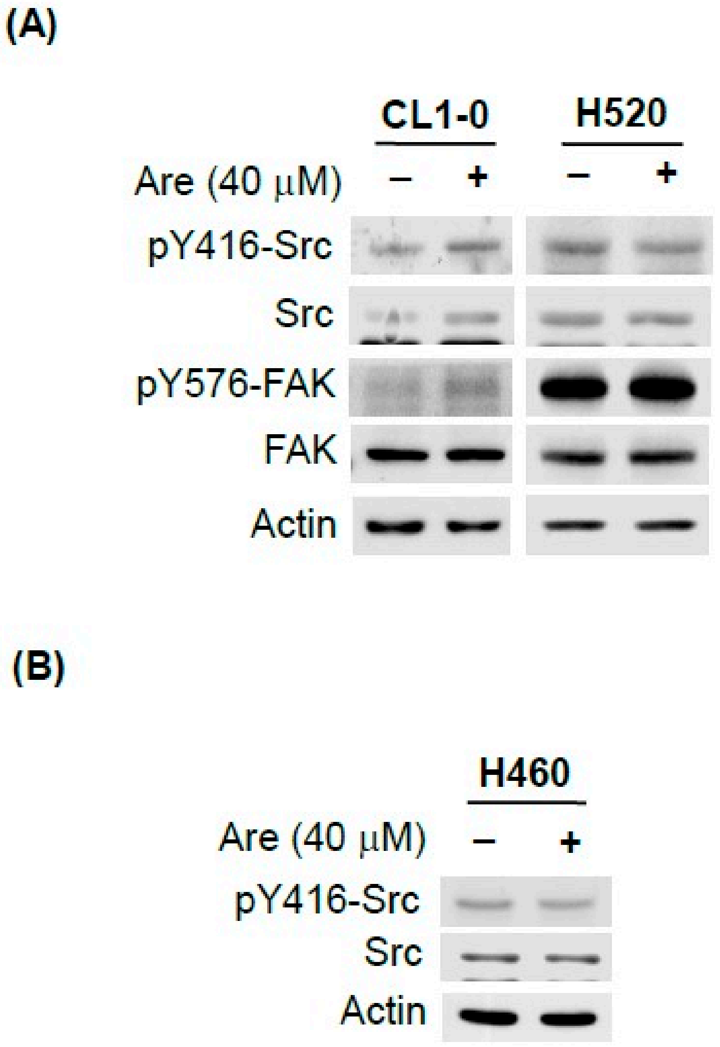

Figure S3. The effects of Arecoline on Src and FAK in CL1-0, H520 (**A**), and H460 cells (**B**). After treated with 40 μM Arecoline for 24 hours, cells were collected and proteins were analyzed by immunoblotting. The expression and phosphorylations of c-Src (p-Y416-Src) and FAK (p-Y397-FAK) as well as total Src/FAK proteins were measured. Actin served as the internal control.
